# Patient-Reported oral health outcome measurement for children and adolescents

**DOI:** 10.1186/s12903-016-0293-x

**Published:** 2016-09-15

**Authors:** Honghu Liu, Ron D. Hays, Marvin Marcus, Ian Coulter, Carl Maida, Francisco Ramos-Gomez, Jie Shen, Yan Wang, Vladimir Spolsky, Steve Lee, Li Cai, James Crall

**Affiliations:** 1 Division of Public Health & Community Dentistry, School of Dentistry, University of California, Los Angeles (UCLA), Los Angeles, CA USA; 2 UCLA Department of Medicine, David Geffen School of Medicine, Los Angeles, CA USA; 3 UCLA Section of Pediatric Dentistry, Los Angeles, CA USA; 4 UCLA Department of Biostatistics, Los Angeles, CA USA; 5 UCLA Section of Restorative Dentistry, Los Angeles, CA USA; 6 UCLA Department of Education, School of Education and Information Studies, Los Angeles, CA USA

**Keywords:** Oral health, Children, Adolescent, Longitudinal survey, Patient-reported outcomes

## Abstract

**Background:**

Oral health is an important component of daily functioning and well-being. A comprehensive patient-reported oral health measure is needed to gauge the impact of oral health status on children and adolescents. This study aims to develop oral health item banks and associated short-form surveys for children and adolescents 2–17 year olds.

**Methods:**

Using children and adolescents, ages 2–17 years, selected from diverse dental sites in Greater Los Angeles Area, we propose to develop state-of-the-science methods to create oral health item banks to effectively measure oral health outcomes for children and adolescents. Methods include a literature review of existing measures, focus groups, cognitive interviews, drafting and field testing of survey items, and evaluation of the psychometric properties of the measures.

**Results:**

Based on the systematic literature search and focus groups, we identified core (physical health, mental health, and social function domains) and peripheral (e.g., need and access) oral health domains. We then drafted survey items and revised them based on 66 cognitive interviews (27 children/adolescents and 39 parents) with 39 families. The revised items will be administered in a field test of 500 children and adolescents ages 2–17, and their parents.

**Conclusions:**

The qualitative methods used in the initial phases of the project (focus group and cognitive interviews) are the initial steps in the development of oral health item banks and associated short-form surveys for children and adolescents. The oral health items can potentially be used to create effective computerized adaptive test and/or create ad hoc short forms targeting specific areas of oral health to survey large populations of children with much less cost compared with traditional clinical oral health examination.

## Background

Oral health is an important component of daily functioning and well-being. Healthy People 2020 [1] notes the importance of prevention and control of oral and craniofacial diseases, conditions, and injuries, and of enhancing access to preventive services and dental care. The 2000 Surgeon General's Report on Oral Health in America [2] showed that early childhood caries is one of the most serious and costly health conditions among young children. The need for oral health care is the most prevalent unmet healthcare need among children and adolescents [3]. Pediatric oral disorders have a negative effect on children’s health-related quality of life [4]. The adolescent years could be a difficult emotional period when dental and medical needs may be neglected [5]. Understanding and assessing children and adolescents is complex as they are not a stable target, rather in the stage of emerging-developmental skills and functions.

Patient-reported outcomes (PROs) are important for assessing oral health status and evaluating the impact of dental care. Prior researches have shown that children 8 years and older are mature enough to be able to accurately self-report health issues [6–8], and respond appropriately to questions regarding their functioning and well-being [9]. More recently, Tsakos et al. (2012)’s study has shown that children as young as 5 years old can provide accurate reports of the impact of oral disorders on their quality of life [10].

Obtaining PROs directly from children, adolescents, and their parents is possible; however, effective assessment requires consideration of developmental issues such as the rapid change in children’s cognitive capacities. Self-reports are the primary method of assessment, supplemented by parent or guardian proxy report when a child is unable to accurately self-report (e.g., too young) [11]. The Patient-Reported Outcome Measurement Information System (PROMIS®) was initiated in 2004 to develop and evaluate health-related quality of life measures. The PROMIS Network’s overall goal is to develop a publicly available set of standardized instruments for measuring major self-reported health domains that are affected by many chronic illnesses [12].

Several questionnaires for measures of oral health-related quality of life have been produced for use with children or using parents as proxies. These generic questionnaires are designed to cover a variety of oral conditions such as dental caries, malocclusion and craniofacial anomalies. The most frequently used measures for self-completion by children are the Child Perceptions Questionnaire (CPQ) [13–15], Child Oral Impacts on Daily Performances Index (C-OIDP) [16], Child Oral Health Impact Profile (COHIP) [17], and PedsQL Oral Health Scale.

This paper describes the approaches, methods and process of our study for the creation, administration and evaluation of oral health items for children and adolescents 2–17, selected from diverse dental sites in Greater Los Angeles Area. The PROMIS methodologies and framework were adopted in this study, as detailed below.

## Methods

### Measurement framework

**Fig. 1:**
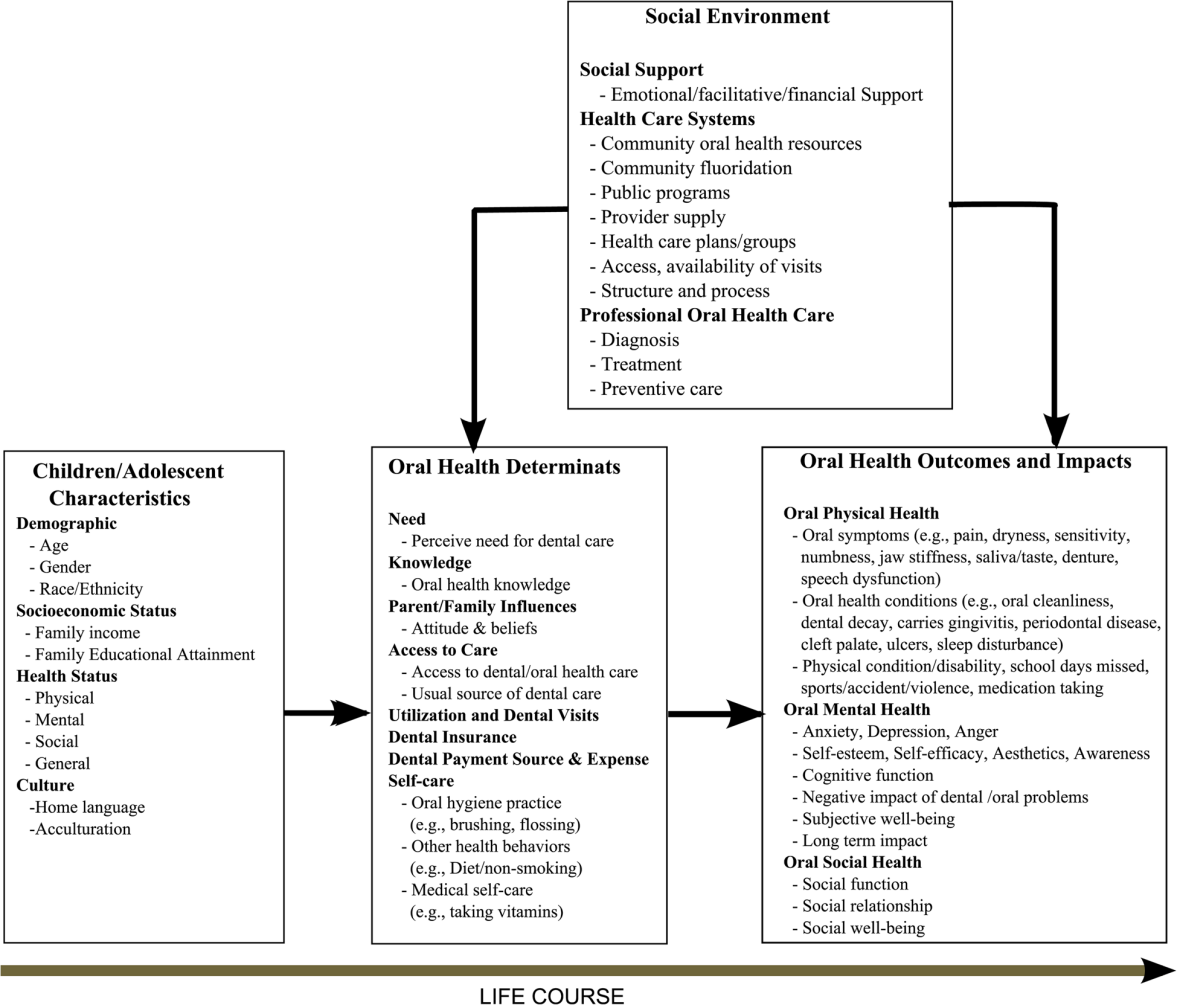
Children/adolescent oral health measurement framework

Our proposed framework for conceptualizing and addressing oral health domains is summarized in Fig. 1. Children and adolescent characteristics have an impact on oral health determinants, which in turn have an impact on oral health outcomes. Social environmental factors also have an impact on both oral health determinants and oral health outcomes. This model builds upon our previous work on the multi-level influences of oral health [18]. It integrates the life-course concept into the dynamics of oral health by including genetic, biological, behavioral, social, and economic contexts that change as a person develops through childhood, adolescence, young adulthood and later adult life [19].

The study has four carefully designed and well-connected operational phases: (1) a systematic review of the literature to identify instruments and survey items associated with oral health; (2) focus groups, cognitive interviews, item selection, and drafting of oral health items; and (3) field testing of subject surveys and dental examinations; and (4) psychometric analyses of oral health items. We have already completed Phase 1 and Phase 2 of the study and are now in the middle of Phase 3.

### PHASE 1: Evaluate the relevance of PROMIS and conduct systematic literature review

We conducted systematic literature searches on existing oral health items previously used to measure oral health, particularly those for children and adolescent ages 2–17, as well as their parents, to capture the latest research, through exhaustive searches of PubMed, Google scholar, the Patient-Reported Outcome and Quality of Life Instruments Database, and the Dental, Oral, and Craniofacial Data Resource Center (DRC). The systematic review and search identified all surveys, instruments and items that have been used to date to measure oral health applicable to children and adolescents, ages 2–17. The DRC serves as a resource on dental, oral, and craniofacial data. We searched through DRC by criteria, which include survey title, acronym, sample design type, sample size, general variables, oral health variables, population, survey type, sponsor, and geographic region. Multiple items can be selected under each criterion. Records can be selected that match all or any of the criteria. Search results included survey overview, sample size, geographic region, population, year, interval, survey type, sample design, method/technique, reliability/validity, general variables, and oral health variables. Key instruments were identified to be considered “legacy” instruments. A modified Delphi approach was used to reach agreement on the domain, sub-domains and elements for oral health within the PROMIS domain hierarchy framework. We adopted the PROMIS method of binning (identifying those items thought to represent distinct domains related to oral health) and winnowing (using a consensus process to exclude items that do not meet the domain definitions or are redundant, etc.), and ensure that the items are concise, simple, have the same item stem, and are targeted to appropriate reading level for the children and adolescents.

### PHASE 2: Focus groups, cognitive interviews, and final item selection

Phase 2 involved convening focus groups with racially/ethnically and geographically diverse groups of children, parents, and oral health professionals, and was conducted to help conceptualize oral health PROs. The cut-off criteria used to separate age groups are based on children and adolescents’ physical, mental and dental development that are pertinent to that specific age group. The basic age breakdown was related to dentition. In the 2–7 age group, children mainly had primary teeth; in the 8–12 age group, there is mixed dentition, including permanent and primary teeth; the 13–17 age group all have permanent teeth. Adolescents were also more likely to have full orthodontic treatment. We conducted focus group interviews with parents of children ages 2–7, 8–13, and 14–17, and with children/adolescents ages 8–13 and 14–17. These focus groups elicited perceptions of oral health in children and adolescents. Each focus group was digitally recorded and transcribed verbatim. The research team conducted thematic and narrative analysis to identify the terms used by the participants.

Following the focus groups, we drafted survey items and administered them in a series of face-to-face cognitive interviews with children and adolescents, parents and professionals. Cognitive interviewing provides a means to evaluate participants’ responses to the items and test their understanding of the meaning of the items. For each candidate item, we probed the subject regarding the item content and response options [20]. We explored how respondents recall information, what time frame they use, and what time frame is beyond their recall. Cognitive interviews were conducted using intermittent and retrospective probes following completion of all items. Based on findings from cognitive interviews, items were then refined for the field test.

### PHASE 3: Field test

The field test collects data to evaluate the oral health items identified through phases 1 and 2. We survey children’s oral health perceptions and conduct dental clinical examination. Children ages 2–7 receive dental clinical examinations only, and their parents completed the survey as their child’s proxy. Children 8–13 and 14–17 received both survey and dental clinical examinations, and their parents also completed surveys. We use a software program called Questionnaire Development System™ (QDS™), developed by Nova Research Company, to build and implement Audio Computer-Assisted Self-Interview Software (ACASI) interviews for child and patient surveys. Each of the surveys contains core items (e.g., demographics and pain) that are applicable to all three age groups and the targeted items that are pertinent only to that specific age group. With the ACASI surveys, each participant hears the same text-to-speech computer-generated voice reading the same instructions and questionnaire items.

Given the large age span of children from 2 to 17, in view of the varying nature of dentation and the level of maturity, we use different versions of the ACASI surveys: one for parents of children ages 2–7, one for children ages 8–17, and one for their parents. Each of the surveys contains core items (e.g., demographics and pain) that are applicable to all three age groups and age-group targeted items.

To evaluate responsiveness to change over time and estimate minimally important differences, a group of 150 randomly selected children and adolescents along with their parents will also complete a follow-up ACASI survey at 6 months from the initial survey to collect information about changes in oral health. They will also be given a follow-up clinical exam at the 6-month period. These children and adolescents will likely have changes, such as loss of primary teeth and body growth, which may affect their aesthetic concept and perception.

### Clinical examination

A dental clinical examination is conducted on all participating children. The oral examination of the 2-7-year group is performed before or after the interview of their parent or caregiver. The clinical examination of the 2–7 year children consists of an examination of the oral mucosa, examination of the teeth for the presence of obvious decay and decalcification (white spots), the presence of plaque on the centrals and molars, when present, and bleeding on probing an inflammation of the gingiva. Oral clinical examinations for children ages 8–17 are performed before or after they have completed the survey. Starting with an examination of the oral mucosa, dental caries are assessed and followed by examination for the presence of plaque, gingivitis and calculus. In addition, the occlusion is assessed as well as orthodontic factors that characterize the position of the teeth, spacing within the arch and facial profile. Condition on tooth crowding, spaces and jaw relationships, such as overbite and overjet, are also noted. Photographs of the profile of front teeth are taken to assess aesthetics and tooth color.

The assessment of the oral mucosa is based on the format developed by the World Health Organization, which consists of a systematic assessment of the lips, labial mucosa and sulcus, commissures, buccal mucosa and sulcus, gingival and alveolar ridges, floor of the mouth, and hard and soft palate. The caries examination consists of a full mouth examination of all primary and permanent teeth. All of the coronal portions are assessed using the DFT/DMFT index, for the primary and permanent dentition, respectively. The measures included in the exam are also consistent with the Children’s Oral Health Status Index [21, 22].

### Sample size

Sample size will be 500 children/adolescents for the field test. The sample will be selected from diverse dental clinics and private practices throughout the Greater Los Angeles Area. With a sample size of 500, the standard error around a correlation is approximately 0.045. A sample size of 500 or more was recommended by Reeve and Fayers (2005) [23] for estimating the item response theory graded response model (2 parameter model).

### Field test recruitment

Four hundred children and adolescents, and their parents will be recruited from community clinics and private practices throughout the Greater Los Angeles Area. These clinics and practices encompass community general clinics, safety-net clinics, group practices, solo general practices, and solo specialty practices with general dentists, pediatric dentists and specialists. These recruitment sites cover different geographic areas and communities, ranging from low-income underserved immigrant neighborhoods to high–income professional communities, with diverse race and ethnic compositions. Institutional review board approval for this study was obtained from the UCLA Office of the Human Research Protection Program.

### Phase 4: Psychometric and statistical analysis

Using data collected from field test, psychometric and statistical analyses will be performed to evaluate oral health items and scales. We will evaluate the reliability and validity of the oral health scales by using item-level factor analytic methods to assess the dimensionality of the oral health scales, and estimate item response theory (IRT) parameters (e.g., threshold, discrimination). Psychometric evaluations will be completed sequentially using the following steps described in Reeve et al.’s paper [24].

### Revision and finalization of item banks

Based on the findings from psychometric and statistical analyses, we will make revisions and adjustments to the selected oral health items to finalize the item banks. For example, we may modify the levels of response for some items or drop the items that do not show good psychometric properties (e.g., very low reliability).

## Results

After comprehensive and systematic literature search on existing oral health items, surveys and instrument relative to children and adolescents, ages 2–17 and their parents, initial core domains for the oral health item banks to be developed are listed in Table 1, which include physical health domains, mental health domains, and social function domains. These initial domains were revised in light of data derived from focus group analysis, cognitive interview, team investigators, experts and analysis.

**Table 1:** Oral health domains

Domains	Measures
Physical Health	Physical Function
	Pain
	Physical Condition
	Sleep Disturbance/Pain
	Other Symptoms
	School Days Missed
	Sports/Accident/Violence
	Medication Taking
	Physical disability
	Function Limitation
	Oral health symptoms
	Halitosis
	Sensory Problem
	Lips/tongue
	Gums/tissues
	Saliva/Taste
	Natural teeth
	Denture
	Oral cleanliness
	Speech function
Mental Health	Anxiety
	Depression
	Anger
	Cognitive Function
	Negative Impact of Dental/Oral Problems
	Awareness
	Self-Esteem
	Self-Efficacy
	Aesthetics
	Sleep disturbance
	Subjective well-being Provided
Social Function	Social Function
	Social Relationship
	Social Well-being

Table 2 lists domains that are related to oral health. Items related to access, behavior, etc., are critical to oral health, but do not yet fit into the current PROMIS framework.

**Table 2:** Oral health related domains

Domains	Measures
Need	Perceived need for dental care
Knowledge	Oral health Knowledge
	Awareness
Health Behavior	Oral hygiene practice
	Smoking
	Alcohol
	Substance abuse
Access	Access to dental/oral health care
	Usual Source of Dental Care
Utilization	Utilization of dental/oral health care
Diet	Diet
Community Level Measures	Community Oral Health Prevention and Intervention
Preventive Dental services	Immunizations, water fluoridation
	Oral health access
	Dental Visits
	Availability of Dentists
Health care delivery	Access, availability of visits
	Process – method by which oral health care is provided
	Outcome – the consequence of the healthcare Provided
	Structure– the environment in which healthcare is provided
Dental Visits	Last dental visits, first dental visits, frequency, type of provider seen.
Dental Insurances	Type of insurance
Dental Expense and Payment Source	Source of payment

Table 3 lists the PROMIS self-reported health domains. These results provided valuable information about core domains for the oral health item banks to be developed and generated content for new items and evaluated with cognitive interviews and in a field test.

**Table 3:** Self-reported health domains in PROMIS

Component	Subcomponent	Domain	Sub-domain
Physical Health	symptoms	Pain	Behavior*
			Interference
			Quality*
			Intensity*
			Impact
		Fatigue	Experience
			Impact
		Asthma Impact	
		Gastro-intestinal Symptoms*	
	Function	Physical Function	Upper Extremity
			Lower Extremity
			Central
			Activities*
			Mobility
		Sexual Function and Satisfaction	Global Satisfaction with Sex Life
			Interest in Sexual Activity
			Lubrication
			Vaginal Discomfort
			Erectile Function
			Orgasm
			Therapeutic Aids
			Sexual Activities
			Anal Discomfort
			Interfering Factors
		Sleep Function	Sleep Disturbance
			Sleep-related Impairment
			Wake Disturbance
		Wake Function	
		Physical Activity	
		Motor	Balance
			Dexterity
			Endurance
			Locomotion
			Strength
		Sensation	Audition
			Olfaction
			Pain
			Taste
			Vestibular
			Vision
Mental Health	Affect	Negative (Emotional Distress)	Anxiety
			Depression
			Anger
			Experience of Stress*
			Psychosocial Illness Impact - Negative
			Meaning & Spirituality
			Stress Response
			Self-concept
			Social Isolation
			Emotional and Behavioral Dyscontrol
			Stigma
			Fear
			Sadness
		Positive	Subjective Well-being*
			Psychosocial Illness Impact - Positive
			Meaning & Spirituality
			Coping
			Self-concept
			Social Connection
			Mastery & control (Self-efficacy)
			Subjective Well-being (positive affect) *
	Behavior	Substance Use/Alcohol	Alcohol Use
			Positive Consequences
			Positive Expectancies
			Negative Consequences
			Negative Expectancies
		Smoking	Nicotine Dependence
			Perceived Benefits: Affective/Hedonic
			Perceived Benefits: Coping
			Perceived Benefits: Social
			Perceived Benefits: Psychosocial
			Perceived Benefits: Health
	Cognition	Applied Cognition - General Concerns	
		Applied Cognition - Abilities	
		Applied Cognition –Attention/ Executive Function	
		Self - efficacy	General
			Self-Efficacy with management of Chronic Disease*
		Episodic memory	
		Language	
		Processing speed	
		Working memory	
Social Health	Relationships	Social Isolation	
		Quality of Social Support	Companionship
			Emotional Support
			Informational Support
			Instrumental Support
			Social Isolation
		Peer Relationships	
			Interaction with peers
		Family Belongingness*	
		Communication	
	Function	Ability to Participate in Social Roles and Activities	Social Roles
			Discretionary Social Activities
		Satisfaction with Participation in Social Roles and Activities	Social Roles
			Discretionary Social Activities

* Under-development as indicated by http://www.assessmentcenter.net/documents/instrumentlibrary.pdf

Through focus groups, we identified three unique themes that the youth associated with their oral health status: (1) understanding the value of maintaining good oral health over the life course, with respect to longevity and quality of life in the adult years; (2) positive association between maintaining good oral health and interpersonal relationships at school, and dating, for older youth; and (3) knowledge of the benefits of orthodontic treatment to appearance and positive self-image, while holding a strong view as to the discomfort associated with braces [25].

We then drafted survey items and revised them based on 66 cognitive interviews (27 children/adolescents and 39 parents) with 39 families. We identified a number of issues from the cognitive interviews that informed subsequent item development. The first issue was guardian involvement in the interview and their reliability as proxies. The second issue was confusing wording of some of the new items created as a result of focus group discussions. The third issue was temporality. Certain items required parents to recall a time when they were concerned about a recent oral health problem or indicate any instances of dental anxiety or phobic reactions. We have completed additional cognitive interviews to test comprehensibility of 23 child/adolescent items and 35 parent items, notably those items that we changed through analysis and expert review of the results of original cognitive interviews, and also to test a few revised legacy items derived from the California Health Interview Survey (CHIS) [26, 27].

## Discussion

Children and adolescents are in the stage of emerging developmental skills and functions. Younger children are already capable of expressing a range of emotions (e.g., anxiety, happiness). However, due to limited level of maturity and responsibilities, children ages 8–17 are not able to answer all questions related to their oral health such as insurance co-pay or other financial questions, for which parents or guardians are the appropriate respondents. Therefore, it is necessary to assess self-reported oral health outcomes for children and adolescents with children surveys supplemented by parent interviews. Furthermore, given the young age of children, oral health outcomes will have profound long-term impacts on their future function and well-being for many years to come, and we need to use life course lens to view the process and impact. Focus groups can serve as an innovative approach to understanding children's experiences from a developmental perspective through children and parents’ involvement.

The National Institute of Health is encouraging use of PROMIS measures in clinical practice and research through the Patient-Centered Assessment Resource: http://healthcaredelivery.cancer.gov/resource/. PROMIS has created an extensive library of item banks, but is still completely lacking oral health item banks. This project capitalized on innovations in modern measurement theory, qualitative methods for instrument development and computerized adaptive testing. In our field testing, we used ACASI approach that has very desirable measurement properties. A research staff is present to instruct the respondent in the use of the laptop computer that administers the questions. The questions are presented both visually on computer screen and audible through earphones so that even respondents with low levels of literacy can take part. ACASI combines the power, flexibility, and standardization of automation with the privacy of self-administration. The audio component of ACASI is particularly effective for participants with low literacy skills. Among the diverse participants and different clinical settings, the majority felt comfortable using the computer, liked the privacy, and faced few difficulties with its use. When they faced difficulties, research staff is present to help solve the technical issues. Weaknesses using ACASI are possible data loss if staff is not properly trained, or the children cannot focus on answering the questions, or the children are cognitively impaired to use the computers. We strived to train research staff and a knowledgeable data manager will be present onsite to troubleshoot so that these issues can be minimized.

Some important future steps are as follows. First, psychometric analyses on the field test data will be performed to construct oral health-targeted items and scales for children and adolescents, ages 2–17. We will use full-information item factor analysis methods [28, 29] to evaluate dimensionality [30–32], calibrate the selected items using the graded response model (GRM) [33], and assess differential item functioning (DIF) using the updated version of Lord’s Wald test [34]. Second, we will estimate the minimally important differences (MID) for the new oral health measures using an anchor-based approach [35]. A subset of 150 patients with longitudinal data will be asked to assess changes in their oral health at second data point compared to baseline (e.g., retrospective global oral health rating: *a lot better, a little better, the same, a little worse*, or *a lot worse*). People who report either getting a “little better” or a “little worse” on oral health will constitute the minimal change subgroup. The change in oral health rating for this group will be used to estimate the MID. The change in oral health self-reports will also be examined using clinical anchors of change (e.g. the difference in number of dental caries between baseline and follow-up by dental exams). Third, linking metrics and cross-walk tables will be created to estimate legacy scores from oral health domains and vice versa, and provide confidence interval around these estimates. Appropriate equity criteria such as the equipercentile equating procedure will be implemented to determine the linking method. Using the information in the item bank library, users will be able to create computerized adaptive tests or short forms tailored to their populations of interest or aspects of oral health (e.g., dental caries issue) of interest. Scores on these tailored measures can then be compared because the items have been mapped onto a common metric.

This study has several limitations. First, our study sample may not be representative of the U.S. general population in that it is comprised of children who are already under dental care and agreed to participate in this project. Second, there are many decisions involved in the evaluation and refinement of item banks. These decisions require extensive skill and experience and are inherently subjective, but the experience of our team will provide a system of checks and balances. Additionally, we will minimize the likelihood of bad decisions by performing a comprehensive set of psychometric and statistical analyses and balance empirical results with clinical input. We are aware of issues that arise when health-related constructs are fit to IRT models (e.g., extreme threshold parameters, very large discrimination parameters, and highly skewed information curves), and will test alternative models in order to find the best fit.

## Conclusions

This project utilizes qualitative and state-of-the-science quantitative methods to develop oral health PROs for children and adolescents. The qualitative methods used in the initial phases of the project (focus group and cognitive interviews) are the initial steps in the development of oral health item banks and associated short-form surveys for children and adolescents. The oral health items can potentially be used to create effective computerized adaptive test and/or create ad hoc short forms targeting specific areas of oral health to survey large populations of children with much less cost compared with traditional clinical oral health examination. These surveys can be used by dentists, oral health researchers and professionals, and public policy makers for oral health screening, program assessment, oral health evaluation with large populations as well as oral health management and policy planning.

## Abbreviations

ACASI: Audio computer-assisted self-interview software
COHIP: Child oral health impact profile
C-OIDP: Child oral impacts on daily performances index
CPQ: Child perceptions questionnaire
DIF: Differential item functioning
GRM: Graded response model
IRT: Item response theory
MID: Minimally important differences
PedsQL™: Pediatric Quality of Life Inventory™
PRO: Patient-reported outcomes
PROMIS: Patient-reported outcomes measurement information system.
